# Supplementary material for: The Systems Biology Research Tool: evolvable open-source software
Source: BMC Syst Biol. 2008 Jun 29;2:55. doi: 10.1186/1752-0509-2-55 (PMC2446383; doi:10.1186/1752-0509-2-55)
Supplement: Additional file 1 — SBRT Archive. An archive of the current version of the Systems Biology Research Tool. [file 1752-0509-2-55-S1.zip › sbrt-1.4.0/doc/developers_guide/api/sbrt/shell/mng/stat/MannWhitneyUManager.html]

MannWhitneyUManager


|  |  |  |
| --- | --- | --- |
| **Overview** \| **Package** \| **Class** \| **Use** \| **Tree** \| **Deprecated** \| **Index** \| **Help** | |  |
| **PREV CLASS**   NEXT CLASS | **FRAMES**    **NO FRAMES**     **All Classes** |  |
| SUMMARY: NESTED \| FIELD \| CONSTR \| METHOD | DETAIL: FIELD \| CONSTR \| METHOD |  |


---


## sbrt.shell.mng.stat Class MannWhitneyUManager

```
java.lang.Object
  sbrt.shell.mng.AbstractProcessManager
      sbrt.shell.mng.stat.MannWhitneyUManager
```

**All Implemented Interfaces:**: KernelProcessManager<java.lang.String,java.lang.String>, ProcessManager<java.lang.String,java.lang.String>

---

``` public final class MannWhitneyUManager extends AbstractProcessManager implements KernelProcessManager<java.lang.String,java.lang.String> ```

This class is used to manage Mann-Whitney tests.

**Author:**
:   This class was written and documented by Jeremiah Wright while in the
    Wagner lab.

---

| **Field Summary** | |
| --- | --- |
| `static UnmodifiableSet<java.lang.String>` | `optionalKeywords`             The set of keywords understood, but not required, by this process manager. |
| `static UnmodifiableSet<java.lang.String>` | `requiredKeywords`             The set of keywords required by this process manager. |
| `static java.lang.String` | `SAMPLE_A_FILE`             The keyword used to denote the name of the file containing sample A. |
| `static java.lang.String` | `SAMPLE_B_FILE`             The keyword used to denote the name of the file containing sample B. |

**Fields inherited from class sbrt.shell.mng.AbstractProcessManager**

`ALGORITHM, COMPLETED, DATA_HEADERS, DEV_NULL, ELAPSED_TIME, FILE_FORMAT, INPUT_FILE, INPUT_FILE_FORMAT, INPUT_FILE_NAME_FILE, ITERATIONS, OUTPUT_FILE_FORMAT, OUTPUT_FILE_NAME, OUTPUT_FILE_NAME_FILE, PERCENT, PERCENT_COMPLETED, SEED, ZERO_CUTOFF`


**Constructor Summary**

`MannWhitneyUManager()`


| **Method Summary** | |
| --- | --- |
| `void` | `execute(java.io.PrintWriter out)`             Executes the process this process manager controls and writes the results to the provided print writer. |
| `UnmodifiableSet<java.lang.String>` | `getOptionalKeys()`             Returns the set of keywords understood, but not required, by this process manager. |
| `MwUAnalysis<java.lang.String>` | `getProcess()`             Returns the process controlled by this process manager. |
| `UnmodifiableSet<java.lang.String>` | `getRequiredKeys()`             Returns the set of keywords required by this process manager. |
| `static java.util.List<java.lang.Double>` | `getSample(java.lang.String fileName)`             Returns the list of values contained in the file with the provided name. |
| `static java.util.List<java.lang.Double>` | `getSample(java.lang.String fileName, java.io.PrintWriter out)`             Returns the list of values contained in the file with the provided name. |
| `void` | `setInput(ManagerInput<java.lang.String,java.lang.String> input, java.io.PrintWriter out)`             Uses the provided input to prepare this process manager for execution. |

**Methods inherited from class sbrt.shell.mng.AbstractProcessManager**

`check, closeFile, closeRm, execute, getFileFormat, getFileFormat, getFileNameSource, getIterations, getSeed, getTextFileFormat, getTextFileFormat, getZeroCutoff, getZeroCutoff, parseInt, parseLong, printProgress, printProgress, printProgress, runProcess, setInput`

**Methods inherited from class java.lang.Object**

`clone, equals, finalize, getClass, hashCode, notify, notifyAll, toString, wait, wait, wait`

**Methods inherited from interface sbrt.shell.mng.ProcessManager**

`check, execute, setInput`

**Field Detail**

### SAMPLE\_A\_FILE

```
public static final java.lang.String SAMPLE_A_FILE
```

:   The keyword used to denote the name of the file containing sample A.

    **See Also:**: Constant Field Values

---


### SAMPLE\_B\_FILE

```
public static final java.lang.String SAMPLE_B_FILE
```

:   The keyword used to denote the name of the file containing sample B.

    **See Also:**: Constant Field Values

---


### requiredKeywords

```
public static final UnmodifiableSet<java.lang.String> requiredKeywords
```

:   The set of keywords required by this process manager.

---


### optionalKeywords

```
public static final UnmodifiableSet<java.lang.String> optionalKeywords
```

:   The set of keywords understood, but not required, by this process
    manager.


**Constructor Detail**

### MannWhitneyUManager

```
public MannWhitneyUManager()
```


**Method Detail**

### getSample

```
public static java.util.List<java.lang.Double> getSample(java.lang.String fileName,
                                                         java.io.PrintWriter out)
```

:   Returns the list of values contained in the file with the provided name.
    The values must be parsable using `DoubleFormatV1`;

    :   **Parameters:**: `fileName` - the name of the file containing a list of values.: `out` - the print writer to which status messages will be printed. **Returns:**: the list of values contained in the file with the provided name.

---


### getSample

```
public static java.util.List<java.lang.Double> getSample(java.lang.String fileName)
```

:   Returns the list of values contained in the file with the provided name.
    The values must be parsable using `DoubleFormatV1`;

    :   **Parameters:**: `fileName` - the name of the file containing a list of values. **Returns:**: the list of values contained in the file with the provided name.

---


### getRequiredKeys

```
public UnmodifiableSet<java.lang.String> getRequiredKeys()
```

:   Returns the set of keywords required by this process manager.

    :   **Specified by:**: `getRequiredKeys` in interface `ProcessManager<java.lang.String,java.lang.String>`
    :   **Returns:**: the set of keywords required by this process manager.

---


### getOptionalKeys

```
public UnmodifiableSet<java.lang.String> getOptionalKeys()
```

:   Returns the set of keywords understood, but not required, by this
    process manager.

    :   **Specified by:**: `getOptionalKeys` in interface `ProcessManager<java.lang.String,java.lang.String>`
    :   **Returns:**: the set of keywords understood, but not required, by this process manager.

---


### getProcess

```
public MwUAnalysis<java.lang.String> getProcess()
```

:   Returns the process controlled by this process manager.

    :   **Specified by:**: `getProcess` in interface `KernelProcessManager<java.lang.String,java.lang.String>`
    :   **Returns:**: the process controlled by this process manager.

---


### setInput

```
public void setInput(ManagerInput<java.lang.String,java.lang.String> input,
                     java.io.PrintWriter out)
```

:   Uses the provided input to prepare this process manager for
    execution. As the input is processed, informative messages are printed to
    the provided print writer.

    :   **Specified by:**: `setInput` in interface `ProcessManager<java.lang.String,java.lang.String>`
    :   **Parameters:**: `input` - all of the information required to prepare this process manager for execution.: `out` - the print writer to which all messages will be printed.

---


### execute

```
public void execute(java.io.PrintWriter out)
```

:   Executes the process this process manager controls and writes the
    results to the provided print writer.

    :   **Specified by:**: `execute` in interface `ProcessManager<java.lang.String,java.lang.String>`
    :   **Parameters:**: `out` - the print writer where the results will be printed. **Throws:**: `java.lang.IllegalStateException` - if no input was provided to this process manager.


---


|  |  |  |
| --- | --- | --- |
| **Overview** \| **Package** \| **Class** \| **Use** \| **Tree** \| **Deprecated** \| **Index** \| **Help** | |  |
| **PREV CLASS**   NEXT CLASS | **FRAMES**    **NO FRAMES**     **All Classes** |  |
| SUMMARY: NESTED \| FIELD \| CONSTR \| METHOD | DETAIL: FIELD \| CONSTR \| METHOD |  |


---
